# Ophthalmia Myology

**Published:** 1902-06

**Authors:** 


### BOOK REVIEWS.

OPHTHALMIA MYOLOGY. A Treatise on the Ocular Muscles. By G. C. Savage, M.D., Nashville, Tenn.

We cannot but admire the rare gift of an analytical and scientific mind as possessed by Dr. Savage. Few men are so gifted as he is, to work out any mathematical and philosophical problem. This knowledge of physiological optics has never been better exemplified than is seen in the present volume. To hear Dr. Savage discuss, at a medical meeting, the various forms of heterophoria is like listening to an adult repeating the alphabet—so thoroughly familiar does he seem with every phase of the subject. Then, again, this comes not only from the rare gift of the analytical mind possessed by the author, but also from the large amount of study which he has given to the subject.

The reviewer does not feel himself competent to do justice to the above work in a minute criticism of the same. Certainly the work is an exhaustive study upon a subject which is given such slight importance in the various modern text-books on ophthalmology. Dr. Savage might be classed as one of the most enthusiastic exponents of the importance played by muscular anomalies in producing various ocular disturbances, and yet, there are many most excellent ophthalmologists who do not attribute such importance to these muscular troubles.

The fact is, we doubt exceedingly, the advisability of trying to place the various forms of muscular errors in the eye upon a scientific basis. We firmly believe that there are too many collateral questions involved to ever reduce this subject to scientific accuracy. The advocates of "partial tenotomies" are growing less every day, if we may judge from the remarks made by ophthalmologists at various medical meetings. We have long ago found out that the successful physician, and the more so, the successful ophthalmologist, must be his own teacher. By this we mean that each case must be studied by and for itself, and in the treatment it is not always well to follow out the remedy as laid down in the books. Dr. Savage is certainly to be congratulated upon the forcible manner in which he has presented the subject, and this volume marks a distinct era as being the only book published exclusively upon muscular errors of the eye. R.

